# Recent Advances in Creep Modelling of the Nickel Base Superalloy, Alloy 720Li

**DOI:** 10.3390/ma6031118

**Published:** 2013-03-20

**Authors:** William Harrison, Mark Whittaker, Steve Williams

**Affiliations:** 1 Materials Research Centre, Swansea University, Singleton Park, Swansea SA2 8PP, UK; E-Mail: m.t.whittaker@swansea.ac.uk; 2 Rolls-Royce plc, PO Box 31, Elton Road, Derby DE24 8BJ, UK; E-Mail: steve.williams@rolls-royce.com

**Keywords:** creep, stress rupture, stress relaxation, superalloy

## Abstract

Recent work in the creep field has indicated that the traditional methodologies involving power law equations are not sufficient to describe wide ranging creep behaviour. More recent approaches such as the Wilshire equations however, have shown promise in a wide range of materials, particularly in extrapolation of short term results to long term predictions. In the aerospace industry however, long term creep behaviour is not critical and more focus is required on the prediction of times to specific creep strains. The current paper illustrates the capability of the Wilshire equations to recreate full creep curves in a modern nickel superalloy. Furthermore, a finite-element model based on this method has been shown to accurately predict stress relaxation behaviour allowing more accurate component lifing.

## 1. Introduction

Nickel-base superalloys perform a key role in gas turbine aero engines due to their superior mechanical properties at elevated temperatures and good corrosion resistance. This high temperature strength is usually provided by a distribution of γ’(Ni_3_(Al,Ti)) precipitates which hinder dislocation movement. The polycrystalline superalloy, Alloy 720Li, is an example of a workable alloy used in the manufacture of turbine discs. In this case a heat treatment procedure has been devised to provide a microstructure consisting of primary γ’(1–10 μm), secondary γ’(70–120 nm) and tertiary γ’(5–50 nm depending on whether the material is quenched or aged).

Turbine components are subjected to high temperatures and high centripetal force which limit their life. However, because they are safety critical, it is imperative that accurate predictions can be made for the safe operating lives of these components. Whilst the operating life of the disc will be fatigue dominated, in order to affect an accurate fatigue life prediction it is critical that the redistribution of stresses at high temperatures be well understood. This stress redistribution will be as a result of creep deformation at the high temperatures experienced in the component, particularly at stress raising features such as blade loading slots, which experience the highest temperatures due to their proximity to the disc rim, and hence the hot gas stream.

Until recent years, creep models have been based on the traditional assumptions of creep deformation, in particular the accuracy of power law type approaches and the application of fracture mechanism maps defining regimes where specific deformation types (*i.e.*, dislocation or diffusional creep) occur. However, the limitations of these methods have been exposed, with concerns raised particularly about issues such as power law breakdown at high stresses and the lack of accuracy of power law techniques such as the Larson-Miller approach [1].

More recent techniques such as the Theta projection method [2,3], Hyperbolic tangent [4] and Wilshire equations [5,6,7,8,9] have offered alternative approaches, with the hyperbolic tangent and Wilshire equations methods showing particular aptitude in fitting stress-rupture data.

In particular the Wilshire equations require further investigation due to their wide range of early successes. These include accurate predictive methodologies for copper [5], aluminium alloys [6] and a wide range of steels [7,8,9]. Also, more recently a process of full creep curve derivation based on numerical analysis of selected times to strain [10,11] has shown promise as an improvement on the Theta projection curve fitting technique. The equations also benefit from close correspondence of observed trends with physical phenomena,* i.e.*, breaks seen in the equations regularly occurring at the yield stress of the material. Such correlations add confidence to the technique and aid in the application of the equations to a wide range of materials.

In the aerospace sector, it is the focus on accurate modelling of the creep curve and in particular times to critical strains which is considered most necessary, since extrapolation of data to long lives is irrelevant given the dominance of fatigue processes over this time period. The current submission offers an interesting perspective of the applicability of the Wilshire equations, since their application to precipitate hardened superalloys has been limited, and certainly creep curve shape has not been previously considered. 

## 2. Experimental Data 

Alloy 720Li (Low interstitial) was developed for gas turbine disc components by modifying the chemical composition of Alloy 720 [12]. The more recent Alloy 720Li has reduced Cr content to suppress sigma phase formation and C and B contents are reduced to improve forgeability. The chemical composition is of the alloy was (wt%) 16.3Cr, 14.7Co, 3.00Mo, 1.31W, 5.02Ti, 2.57Al, 0.011C, 0.026Zr, 0.015B with balance Ni.

**Figure 1:**
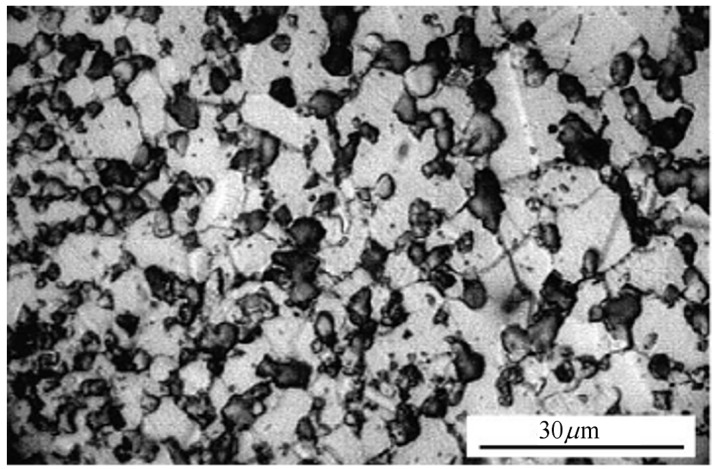
Microstructure of Alloy 720Li.

In order to produce a homogenous microstructure of γ’, standard thermomechanical treatments were used. Post heat treatment, average primary, secondary and tertiary γ’ particles sizes of 5 μm, 100 nm and <10 nm respectively were present. The alloy had a total volume fraction of γ’ particles of ~30%. The resultant microstructure of the material, shown in Figure 1 had a grain size of approximately 10 μm.

Creep tests were performed at constant stress at a range of temperatures between 550 °C and 750 °C with applied stresses chosen to give rupture lives from 5 h to 5,000 h. Specimen strain was recorded for the duration of each test. A stress relaxation test was completed at 650 °C in air [13]. For this test, specimen strain was held at 0.5% for 20 h, followed by an increase in strain to 0.7% for a further 20 h. Specimen load was monitored throughout the test.

Tensile strength, *σ*_TS_, values were obtained from tensile tests at each creep temperature (550–750 °C). These tests were performed under strain control using a constant strain rate of 5 × 10^−5^ s^−1^ increasing upon yielding to a faster rate of ~0.002 s^−1^ until failure.

## 3. Results and Discussion 

**Figure 2:**
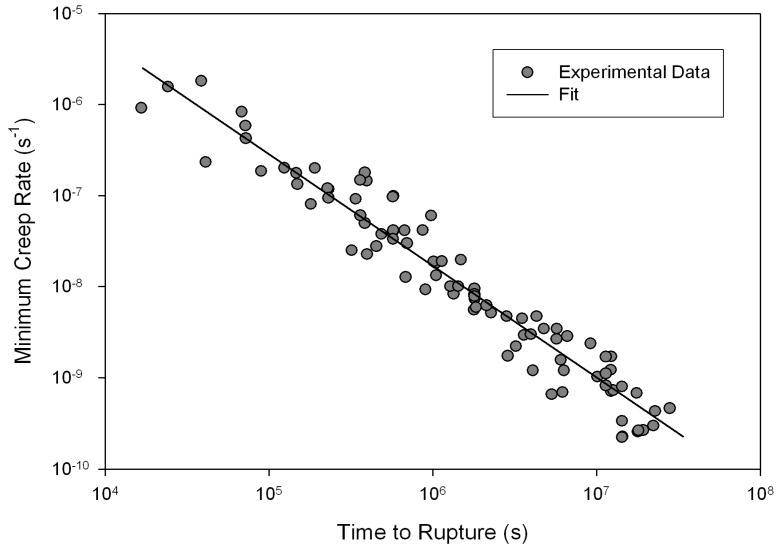
Relationship of minimum creep rate (${\dot{\varepsilon }}_{\text{m}}$) with rupture time ($t_{\text{f}}$) for Alloy 720Li (550 °C to 750 °C).

For all creep tests, normal creep curves were recorded. On loading, an initial strain ($\varepsilon_{0}$) is observed, followed by primary creep where creep rate ($\dot{\varepsilon }=\text{d}\varepsilon /\text{d}t$) decreases until a minimum value is obtained (${\dot{\varepsilon }}_{\text{m}}$). After ${\dot{\varepsilon }}_{\text{m}}$, the creep rate accelerates during tertiary creep until failure. Rupture time ($t_{\text{f}}$) and strain to failure ($\varepsilon_{\text{f}}$) were recorded and utilised in the plotting of traditional creep data analysis. Rupture time decreases with increasing ${\dot{\varepsilon }}_{\text{m}}$ (Figure 2) and the data can be represented using the Monkman-Grant relation [14]

(1) $$M=t_{\text{f}}{\dot{\varepsilon }}_{\text{m}}$$

where the product, *M*, is the Monkman-Grant constant. For Alloy 720Li a value of M≅0.272 was obtained. 

In line with common practice, and using the data shown in Figure 2, the stress (σ) and temperature (T) dependence of ${\dot{\varepsilon }}_{\text{m}}$ and $t_{\text{f}}$ can be calculated using the power law

(2) $$\frac{M}{t_{\text{f}}}={\dot{\varepsilon }}_{\text{m}}=A\sigma^{n}\exp\left(\frac{-Q_{c}}{RT}\right)$$

where *R* = 8.314 Jmol^−1^K^−1^. *A* and *n* are material parameters dependent on stress and temperature. The stress exponent *n* varies from n≅14 for the tests with conditions giving short lives to n≅5 for the long duration tests. $Q_{c}$ is the activation energy for creep with a value of >400 kJ mol^−1^. However, this value also varies with test conditions. The stress and temperature dependence of ${\dot{\varepsilon }}_{\text{m}}$ and $t_{\text{f}}$ can be displayed on single curve by normalising *σ* by the ultimate tensile stress *σ*_TS_ of the material. Therefore Equation (2) becomes

(3) $$\frac{M}{t_{\text{f}}}={\dot{\varepsilon }}_{\text{m}}=A^{*}{\left(\frac{\sigma }{\sigma_{\text{TS}}}\right)}^{n}\exp\left(\frac{-Q_{c}^{*}}{RT}\right)$$

where $A^{*}\neq A$ and $Q_{c}^{*}\neq Q_{c}$; $Q_{c}^{*}$ is lower than $Q_{c}$ since $\sigma_{\text{TS}}$ partially allows for the dependence of creep properties on temperature. For Alloy 720Li, a value of $Q_{c}^{*}$ ≅ 330 kJ mol^−1^ was established. Values for $\sigma_{\text{TS}}$ obtained from tensile test data at each creep temperature along with values of 0.2% proof stress, $\sigma_{\text{Y}}$ [15] are shown in Table 1.

**Table 1:** Tensile properties of Alloy 720Li (550 °C to 750 °C).

Temperature (°C)	$\sigma_{\text{TS}}$ (MPa)	$\sigma_{\text{Y}}$ (MPa)
550	1500	1088
600	1468	1067
650	1420	1017
700	1254	927
750	1084	787

**Figure 3:**
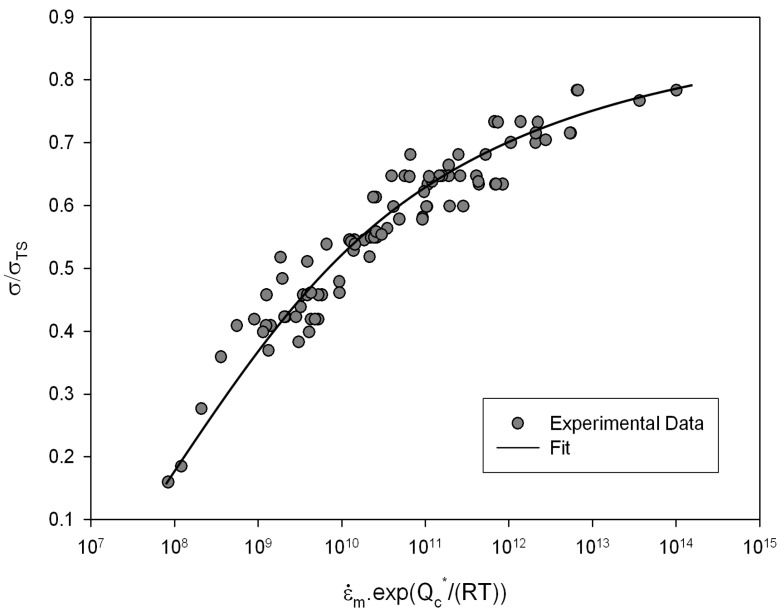
Power Law representation of Alloy 720Li (550 °C to 750 °C).

The dependence of temperature compensated ${\dot{\varepsilon }}_{\text{m}}$ with $\sigma /\sigma_{\text{TS}}$ is shown in Figure 3. This plot shows that *n* in Equation (3) can not be considered constant and varies with applied σ. The variability in *n* values has been attributed to varying creep mechanisms. This inconsistency in *n* makes predicting creep properties over a wide range of test conditions difficult. For example the ability to predict long term creep properties from short term test data is very important in reducing the duration of component design, and is essentially impossible since no extrapolation can be robustly predicted due to the unknown curvature of the graph. Other methods have attempted to address this issue, such as the Wilshire equations [5,6,7,8,9], which have proved effective in extrapolation of creep properties over a range of creep conditions.

### 3.1. Extrapolation of Creep Data

**Figure 4:**
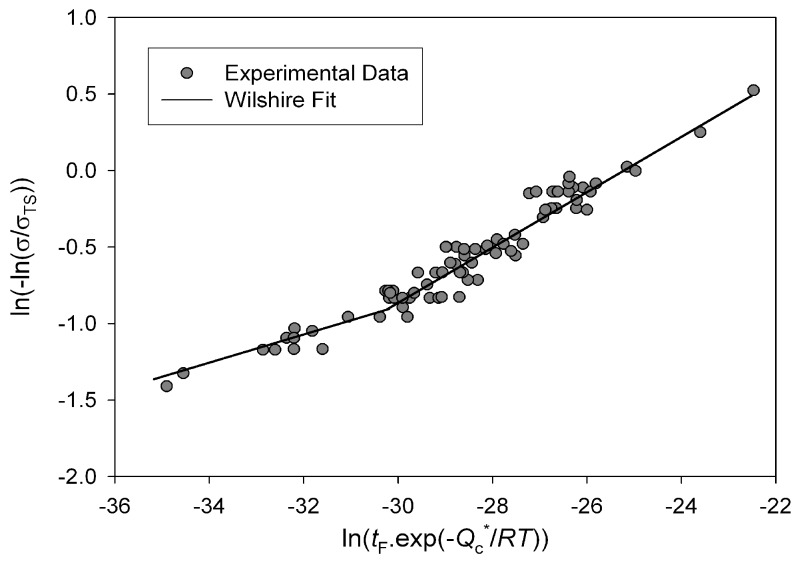
Dependence of $\ln\left(-\ln\left(\sigma /\sigma_{\text{TS}}\right)\right)$ on $\ln\left(t_{\text{f}}\exp\left(-Q_{c}/RT\right)\right)$ for Alloy 720Li, with $Q_{c}^{*}$ ≅ 330 kJ mol^−1^.

**Figure 5:**
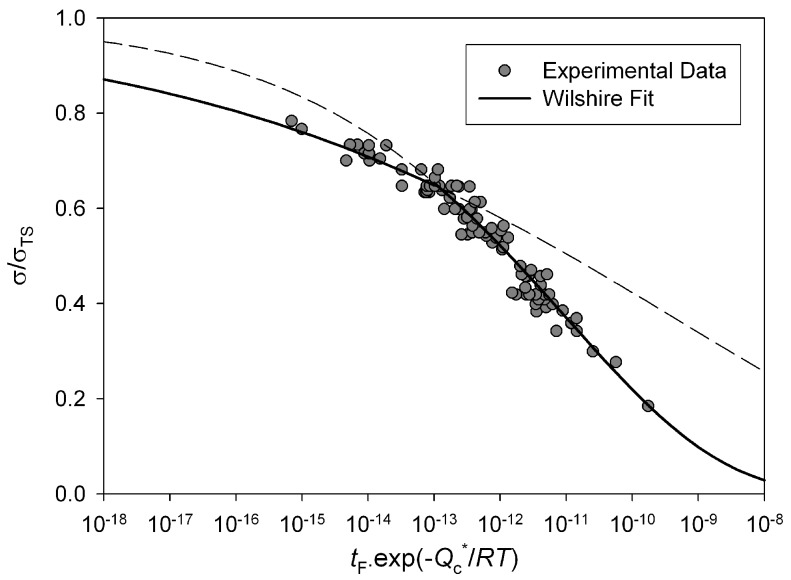
Stress rupture plots for Alloy 720Li with stress normalised by $\sigma_{\text{TS}}$ predicted using Equation (4) (550 °C to 750 °C).

**Figure 6:**
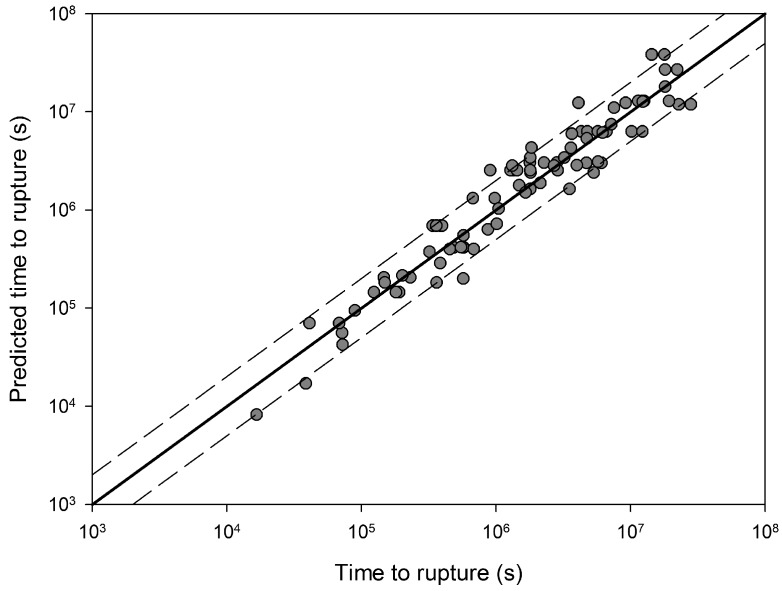
A comparison between experimental creep rupture lives for Alloy 720Li and those obtained using Equation (4) (550 °C to 750 °C). Dashed lines represent a deviation of a factor +/−2.

For any material, applying a stress equal to *σ*_TS_ at any given temperature will result in near instantaneous rupture. Furthermore, creep rupture cannot be expected if the applied stress is zero. Therefore creep properties must be considered over the range $0\leq \sigma \leq \sigma_{\text{TS}}$. Creep prediction methods must allow for the fact that ${\dot{\varepsilon }}_{\text{m}}\to \infty$ and $t_{\text{f}}\to 0$ as $\left(\sigma /\sigma_{\text{TS}}\right)\to 1$. Similarly, the following must be true, as $\left(\sigma /\sigma_{\text{TS}}\right)\to 0$, ${\dot{\varepsilon }}_{\text{m}}\to 0$ and $t_{\text{f}}\to \infty$. The Wilshire equations make use of the sigmoidal shape of the function $f\left(\sigma \right)=\ln\left(-\ln\left(\sigma /\sigma_{\text{TS}}\right)\right)$ and have been shown to accurately predict creep properties over a range of creep conditions. These equations relate temperature compensated $t_{\text{f}}$ and ${\dot{\varepsilon }}_{\text{m}}$ to $\sigma /\sigma_{\text{TS}}$ using

(4) $$\frac{\sigma }{\sigma_{\text{TS}}}=\exp\left\{-k_{1}{\left[t_{\text{f}}\exp\left(\frac{-Q_{\text{c}}^{*}}{RT}\right)\right]}^{u}\right\}$$

and

(5) $$\frac{\sigma }{\sigma_{\text{TS}}}=\exp\left\{-k_{2}{\left[{\dot{\varepsilon }}_{\text{m}}\exp\left(\frac{Q_{\text{c}}^{*}}{RT}\right)\right]}^{v}\right\}$$

respectively. The activation energy for creep ($Q_{c}^{*}$) for the Wilshire equations is equivalent to that obtained for the power law Equation (3) where $Q_{c}^{*}$ ≅ 330 kJ mol^−1^; *u*, *k*_1_, *v* and *k*_2_ are material constants obtained from experimental data. Plotting $\ln\left(-\ln\left(\sigma /\sigma_{\text{TS}}\right)\right)$ against the natural logarithm of temperature compensated rupture times (ln(*t*_f_ exp (−*Q_c_*/*RT*)) gives a straight line of gradient *u* and y-axis intercept of ln*k*_1_ (Figure 4). For Alloy 720Li a different set of *u* and *k*_1_ values were obtained above and below $\sigma /\sigma_{\text{TS}}≅0.65$ indicated by the lines in Figure 4. This value is similar to the yield stress of the alloy and is consistent with behaviour observed in other creep resistant alloys [4,5,6,7,8]. The values of *u* and *k*_1_ obtained from stresses corresponding to *σ*/*σ*_TS_ < 0.65 were 0.184 and 105.53 respectively whereas at stresses greater than the break point values of 0.0993 and 8.471 respectively were obtained (Table 1). These values produce stress rupture master curves which represent experimental behaviour over a range of conditions (Figure 5). The quality of fit can be displayed by plotting creep lives predicted using the above method to those observed experimentally (Figure 6). This figure shows a good quality fit over a wide range of test lives.

**Figure 7:**
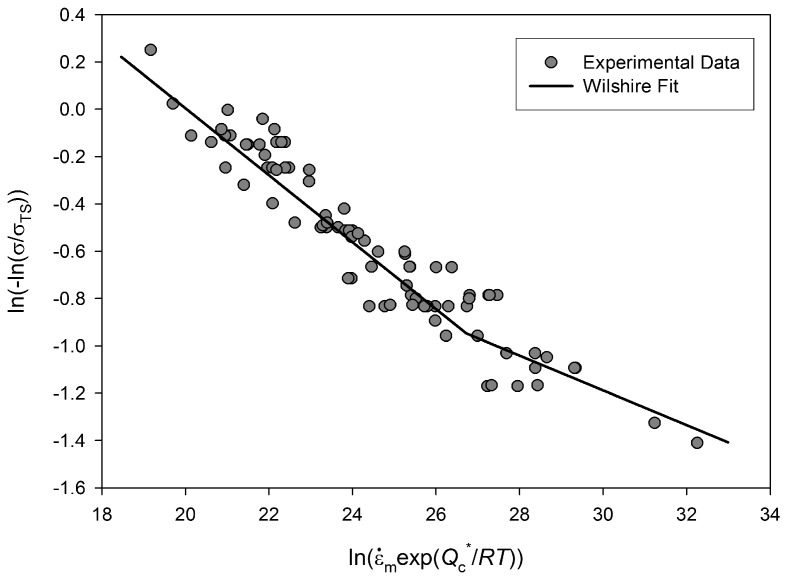
Dependence of $\ln\left(-\ln\left(\sigma /\sigma_{\text{TS}}\right)\right)$ on $\ln\left({\dot{\varepsilon }}_{\text{m}}\exp\left(-Q_{c}/RT\right)\right)$ for Alloy 720Li, with $Q_{c}^{*}$ ≅ 330 kJ mol^−1^.

**Figure 8:**
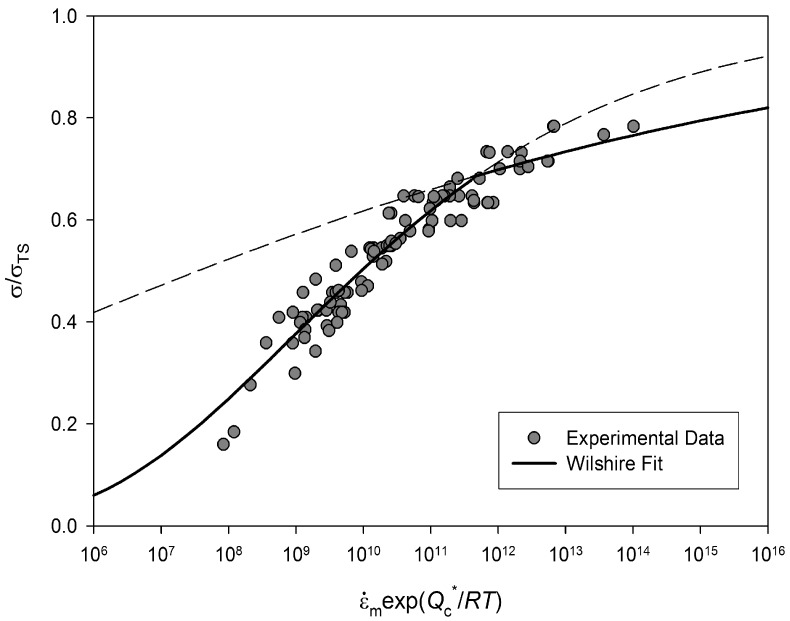
Minimum creep rate plots for Alloy 720Li with stress normalised by $\sigma_{\text{TS}}$. Solid lines represent the fit obtained using Equation (5) (550 °C to 750 °C).

**Figure 9:**
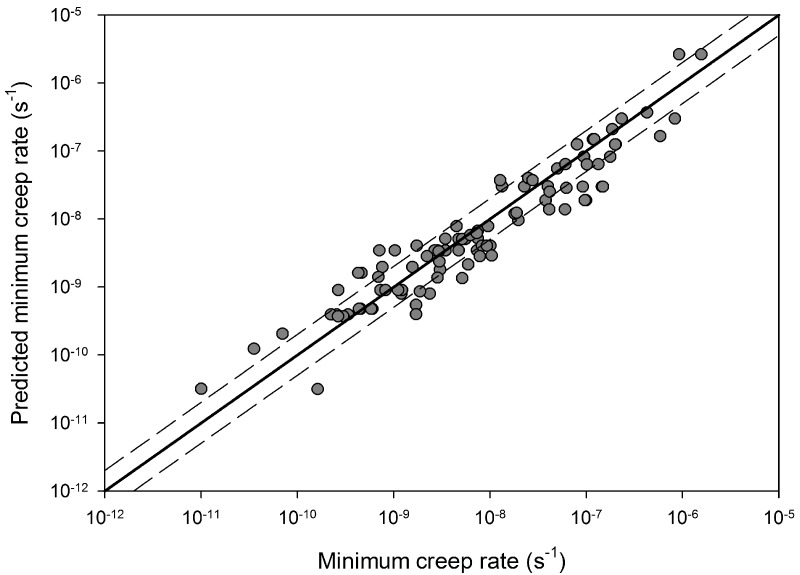
A comparison between experimental minimum creep rates for Alloy 720Li and those obtained using Equation (5) (550 °C to 750 °C). Dashed lines represent a deviation of a factor +/−2.

A similar approach is used for predicting minimum creep rates. *v* and *k*_2_ are obtained from the gradient and intercept of a best fit line through a plot of $\ln\left(-\ln\left(\sigma /\sigma_{\text{TS}}\right)\right)$ against $\ln\left({\dot{\varepsilon }}_{\text{m}}\exp\left(-Q_{c}/RT\right)\right)$ (Figure 7). Again, a change in the value of the coefficients is observed above and below $\sigma /\sigma_{\text{TS}}≅0.65$. At stresses corresponding to $\sigma /\sigma_{\text{TS}}<0.65$, values of *v* = −0.154 and *k*_2_ = 23.58 are obtained. At stresses corresponding to $\sigma /\sigma_{\text{TS}}>0.65$, values of *u* = −0.064 and *k*_2_ = 2.223 are obtained (Table 1). Figure 8 shows minimum creep rates predicted using this method and Figure 9 illustrates that once more, this method gives a good quality representation of the test data.

### 3.2. Creep Deformation 

**Figure 10:**
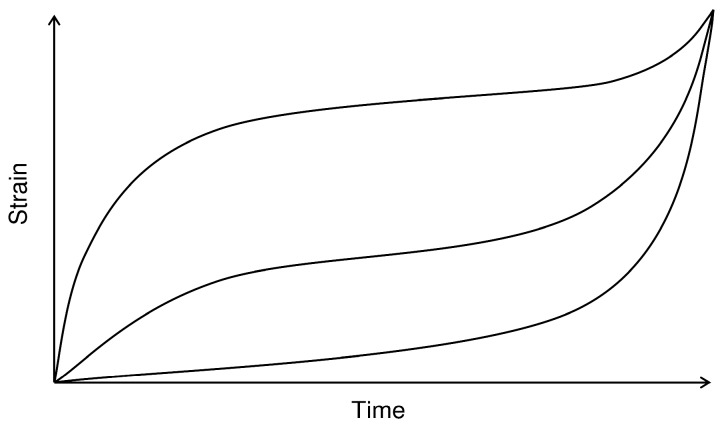
Schematic representation of different creep curves with equal values of ${\dot{\varepsilon }}_{\text{m}}$, $\varepsilon_{\text{f}}$ and $t_{\text{f}}$.

The equations above represent a current method for extrapolating $t_{\text{f}}$ and ${\dot{\varepsilon }}_{\text{m}}$. However an understanding of these values alone is not sufficient for component design since they do not quantify the full shape of a creep curve. This is illustrated in Figure 10 which shows 3 distinct creep curves with identical values of ${\dot{\varepsilon }}_{\text{m}}$, $\varepsilon_{\text{f}}$ and $t_{\text{f}}$. For design purposes it is often necessary to calculate time to a given strain or to predict stress relaxation, both of which require a method capable of predicting creep curve shapes over a wide range of stress and temperature. 

**Figure 11:**
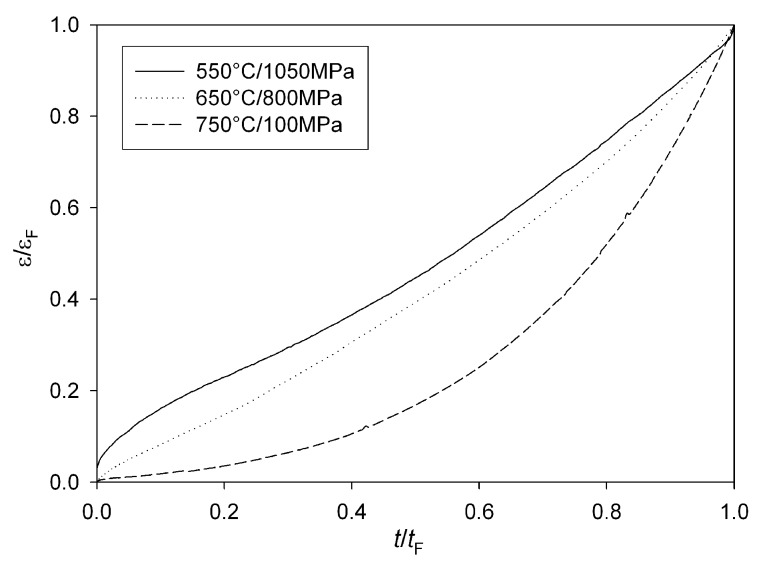
Normalised creep curves showing the variation in the proportion of primary to tertiary creep with test conditions.

The creep curves obtained for Alloy 720Li all display a period of primary creep where creep rate decreases subsequent to loading, followed by tertiary creep where creep rate accelerates to failure, however the proportion of primary to tertiary creep varies with applied test conditions (Figure 11).

The creep curves demonstrate a smooth transition from primary to tertiary creep consistent with the model proposed by Evans [2,3] as opposed to “steady state” creep models with associated secondary creep. Although these models can be used to analyse creep curve shapes, there is a need to quantify creep curve shape. Each creep curve, after removing the strain on loading, may be broken down into primary creep strain, $\varepsilon_{\text{P}}$, tertiary creep strain, $\varepsilon_{\text{T}}$, and strain at failure, $\varepsilon_{\text{F}}$, where

(6) $$\varepsilon_{\text{F}}=\varepsilon_{\text{P}}+\varepsilon_{\text{T}}$$

**Figure 12:**
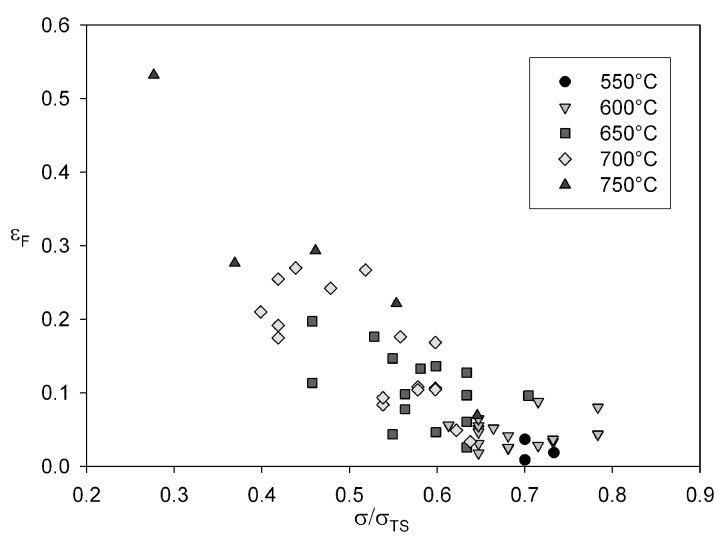
Dependence of strain to failure, $\varepsilon_{\text{F}}$ against normalised stress for Alloy 720Li at 550 °C to 750 °C.

**Figure 13:**
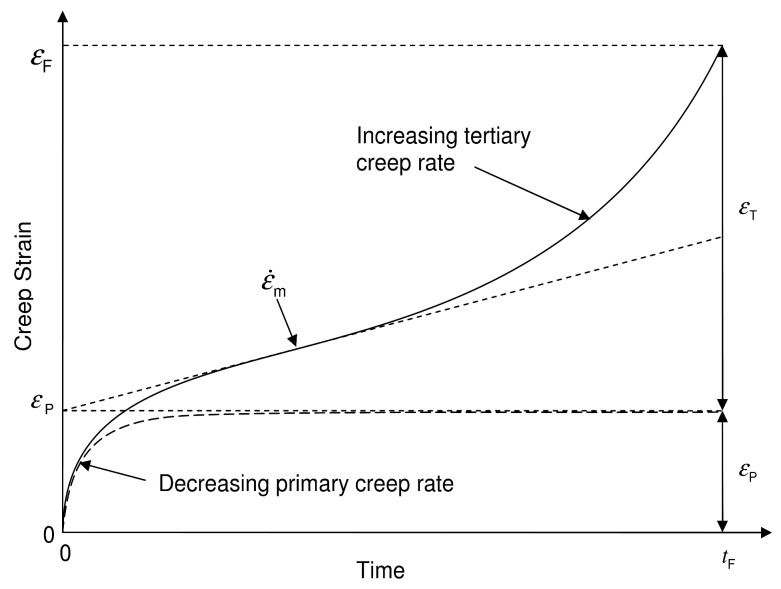
Description of a creep curve using $\varepsilon_{\text{P}}$, ${\dot{\varepsilon }}_{\text{m}}$, $t_{\text{f}}$ and $\varepsilon_{\text{F}}$.

**Figure 14:**
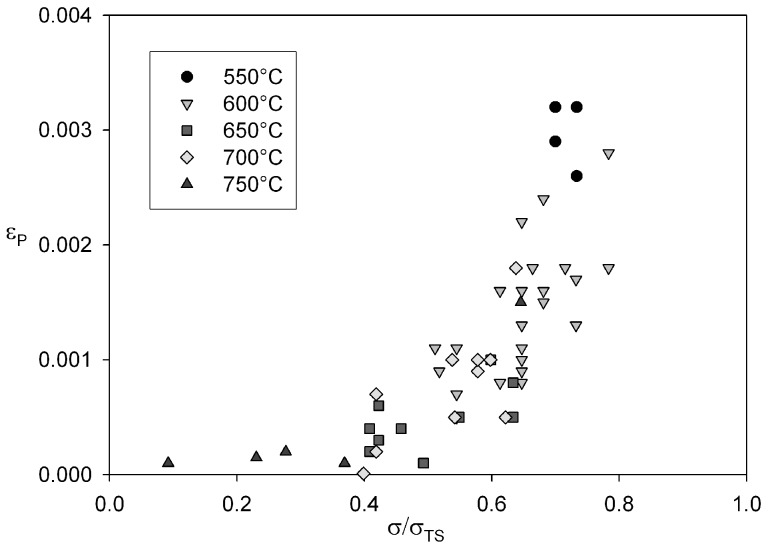
Dependence of primary creep strain, $\varepsilon_{\text{P}}$ against normalised stress for Alloy 720Li at 550 °C to 750 °C.

**Figure 15:**
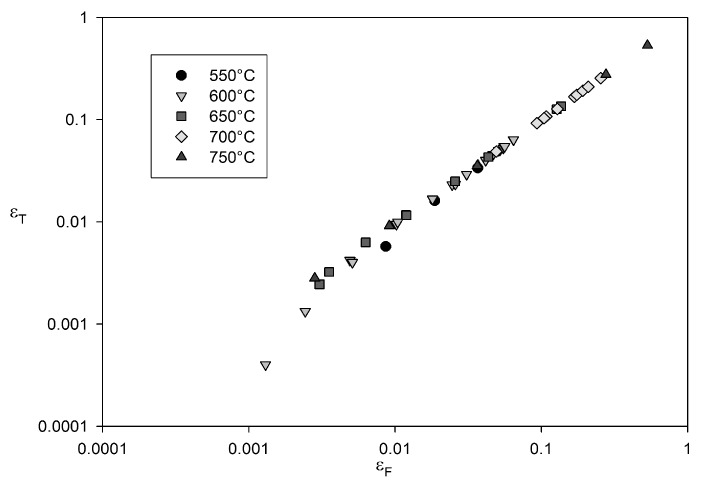
Dependence of total creep strain to failure, $\varepsilon_{\text{F}}$ on tertiary creep strain for Alloy 720Li at 550 °C to 750 °C.

Strain to failure is dependent on applied stress and decreases as $\left(\sigma /\sigma_{\text{TS}}\right)\to 1$ (Figure 12). $\varepsilon_{\text{P}}$ can be approximated by extrapolating ${\dot{\varepsilon }}_{\text{m}}$ back to *t* = 0 (Figure 13). Comparing $\varepsilon_{\text{P}}$ to applied stress helps to quantify the difference in curve shape observed in Figure 11. Below about 700 MPa, the primary component of creep strain is negligible. By plotting $\varepsilon_{\text{P}}$ against stress normalised by tensile stress some of the effects of temperature can be offset (Figure 14). This plot shows that below 0.4$\sigma /\sigma_{\text{TS}}$, $\varepsilon_{\text{P}}$ is negligible and above 0.6*σ/σ*_TS_ this value increases rapidly, however for all tests $\varepsilon_{\text{P}}$ is small compared to *ε*_T_ and hence *ε*_F_* ≈ ε*_T_ (Figure 15). These observations show that creep rupture is dominated by tertiary creep and hence rupture can be characterised by micromechanical deformation and damage processes that occur during tertiary creep in Alloy 720Li.

Many methods have been proposed to predict creep curve shapes from early experiments on fuse wire by Andrade [16] and methods describing creep deformation of pipe flanges proposed by Bailey [17]. Many methods use a function to describe the shape of the creep curve and relate the parameters of this function to applied conditions. The θ-projection method [2,3] is one such technique which extends the work of McVetty [4] and Garofalo [18]. This method uses the function

(7) $$\varepsilon =\theta_{1}\left(1-e^{-\theta_{2}t}\right)+\theta_{3}\left(e^{\theta_{4}t}-1\right)$$

to describe the accumulation of creep strain, *ε*, with time, *t*, where *θ*_1–4_ are material constants dependent on stress and temperature. The parameters *θ*_1_ and *θ*_2 _ describe primary creep and *θ*_3_ and *θ*_4_ represent tertiary creep. The function

(8) $$\log\left(\theta_{\text{n}}\right)=a_{\text{n}}+b_{\text{n}}T+c_{\text{n}}\sigma +d_{\text{n}}\sigma T$$

is then used to evaluate *θ*_1–4_ with stress and temperature where *n* = 1 −4. However, this function is not valid for the full range of creep conditions $0\leq \sigma \leq \sigma_{\text{TS}}$. A constitutive method based on the *θ*- projection method has been formulated to predict creep rate (${\dot{\varepsilon }}_{\text{c}}$) over varying stress and temperature based on evolution of internal material state variables [19]. An alternative method for predicting the evolution of creep rate with time is the continuum damage method proposed by Kachanov [20] and later modified by Rabotnov [21]. Othman and Hayhurst [22] extended this method to account for the effects of primary creep.

### 3.3. Extrapolation of Creep Curves

A more recent approach of predicting creep curve shapes has been proposed based on the Wilshire equations [10,11]. This method consists of relating times to given strains (*t_ε_*) to applied conditions using an equation similar to Equation (4)

(9) $$\frac{\sigma }{\sigma_{\text{TS}}}=\exp\left\{-k_{3}{\left[t_{\varepsilon }\exp\left(\frac{-Q_{\text{c}}^{*}}{RT}\right)\right]}^{w}\right\}$$

**Figure 16:**
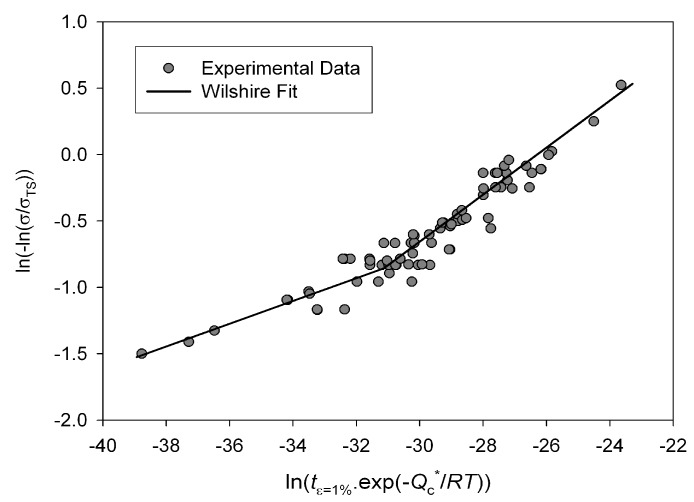
Dependence of $\ln\left(\ln\left(\sigma /\sigma_{TS}\right)\right)$ on $\ln\left(t_{\varepsilon =1\%}\exp\left(-Q_{c}/RT\right)\right)$ for Alloy 720Li, with $Q_{c}^{*}$ ≅ 330 kJ mol^−1^.

*w* and *k*_3_ are material constants dependent on ε and are obtained by plotting ln (ln(*σ*/*σ_TS_*)) against ln (*t_ε_* exp (−*Q_c_*/*RT*)) for times to finite strains using a similar method as used to calculate *u* and *k*_1_ (Figure 16). 

**Figure 17:**
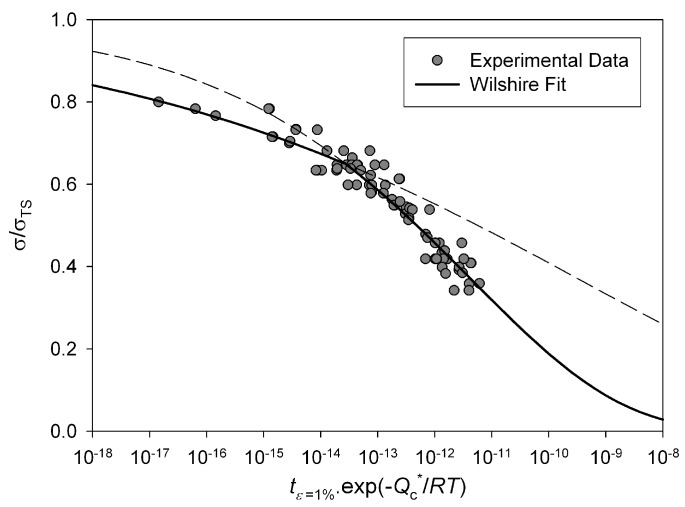
Time to 1% creep strain, *t*_ε_ = 1%, for Alloy720Li predicted using Equations (15) and (16) (550 °C to 750 °C).

**Figure 18:**
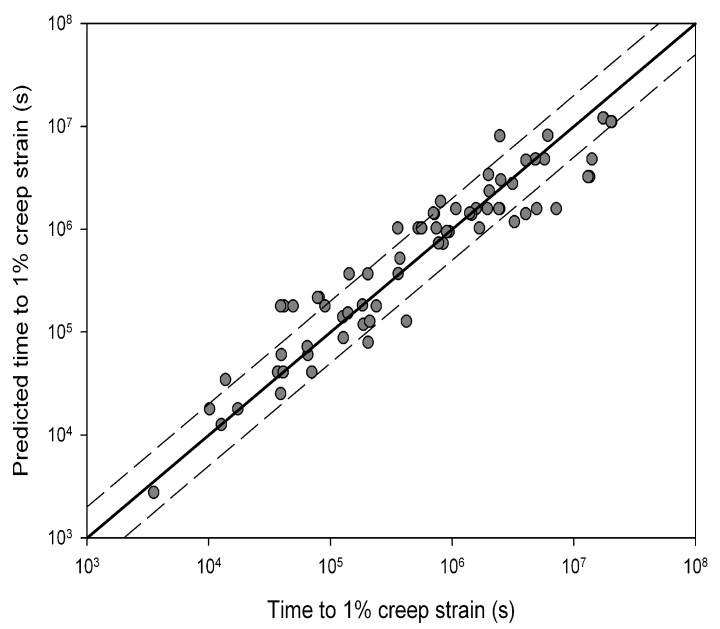
A comparison between experimental times to 1% creep strain for Alloy 720Li and those obtained using Equation (9) (550 °C to 750 °C). Dashed lines represent a deviation of a factor +/−2.

The activation energy used is equal to that found for Equations (4) and (5) ($Q_{c}^{*}$ ≅ 330 kJ mol^−1^). From these values a single value for *w* is chosen to represent all strain levels, and values of *k*_3_ are optimised for each ε using this constant *w*. Relating *k*_3_ to ε allows creep curves to be generated by constructing *t*/ε pairs. Currently, the proposed method relates* k*_3_ to ε using

(10) $$k_{3}=k_{3,0}+k_{3,1}\varepsilon^{-k_{3,2}}$$

where *k*_3,0_, *k*_3,1_ and *k*_3,2_ are material constants. Once the constants are known, Equation (9) can be used to calculate “life to critical strain” and stress/time plots for individual values of creep strain can be calculated. A stress/time plot for 1% creep strain is shown in Figure 17 and the quality of fit is displayed in Figure 18.

**Figure 19:**
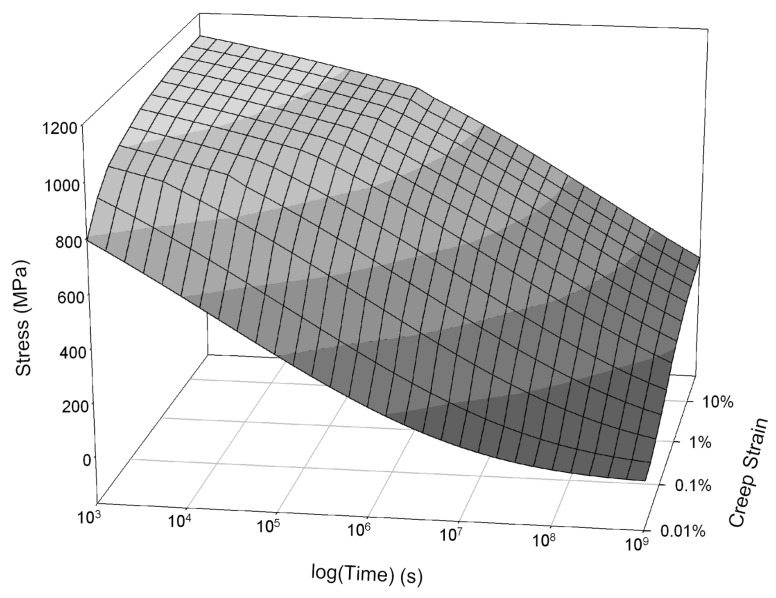
Dependence of creep strain, $\varepsilon_{\text{c}}$, on stress, σ, and time for Alloy 720Li at 650 °C as predicted by the Wilshire creep curve method.

**Figure 20:**
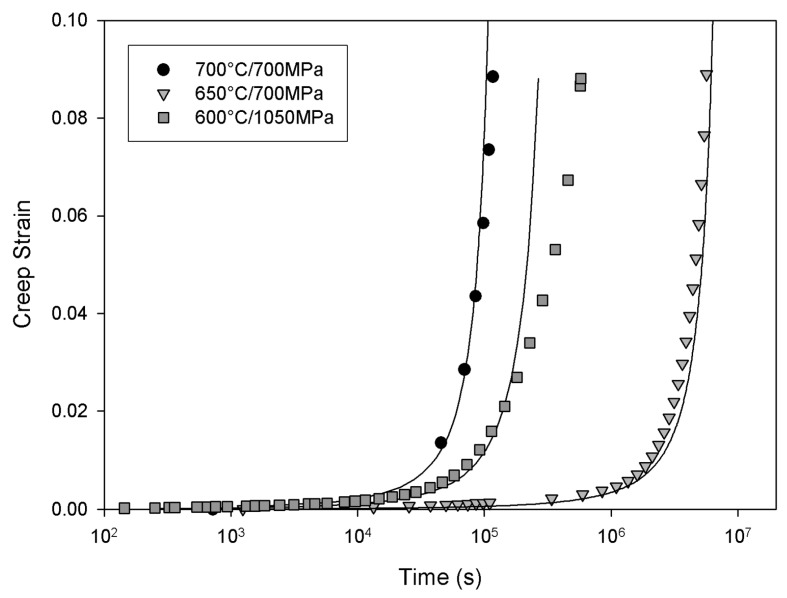
Predicted and experimental creep curves for Alloy 720Li at 650 °C and 700 °C.

Figure 19 shows an isothermal surface displaying the relationship between stress, strain and time predicted by the model. This method can be used to predict whole creep curves over a wide range of conditions. Figure 20 shows a comparison between predicted and experimentally obtained creep curves.

### 3.4. Stress Relaxation

For design purposes the ability to predict creep behaviour in engineering components is essential. One of the ultimate goals of the work was develop a method to accurately predict stress relaxation behaviour, in order to improve the accuracy of fatigue lifing of components based on initial stress conditions. To achieve this, a finite element model was derived based on the aforementioned creep curve method. An important aspect of modelling creep behaviour is accounting for prior creep deformation. Using the method described above, the shape of a creep curve can be accurately predicted for a given applied stress and temperature. The creep rate depends on the position on the creep curve with respect to time (or strain).

Methods used to predict creep behaviour may be based on time hardening, strain hardening or life-fraction hardening. Time fraction hardening is the simplest concept where creep rate is calculated based on the creep rate at time equal to *t*_TOT_, the total analysis time. This method is only applicable to cases where the stress state remains relatively constant. The strain hardening model is more suitable in instances where the stress varies considerably during an analysis, however, this method may produce inaccuracies if the conditions vary from those displaying a primary dominated creep curve to those displaying a tertiary dominated creep curve or *vice versa*. Life-fraction hardening attempts to address this issue by calculating creep rate based on effective time, *t*_EFF_, where *t*_EFF_ = *w*.*t*_F_. *w* is life fraction ratio (*t*/*t*_F_) calculated at the end of each increment. This method has the advantage of predicting creep rupture when *w* exceeds 1.

The creep rate for any given increment can be calculated by the difference in effective creep strain at the beginning and end of the time increment, and the duration of the time increment, Δ*t*

(11) $$\dot{\varepsilon }=\frac{\Delta \varepsilon }{\Delta t}=\frac{\varepsilon_{i+1}-\varepsilon_{i}}{\Delta t}$$

For strain hardening, the magnitude of creep strain at the beginning of the time increment, $\varepsilon_{i}$, is known and so a value of effective time for the beginning of the increment, *t*_i_, can be calculated using Equations (9) and (10). The value of effective time at the end of the increment, $t_{\text{i+1}}$, is simply $t_{\text{i}}+\Delta t$. The value of creep strain at the end of the time increment, $\varepsilon_{i+1}$, cannot be calculated directly from time so an iterative method must be used. For this an initial estimate of $\varepsilon_{i+1}$ is required. For the first increment, this can be set to an arbitrary value ($\varepsilon_{0}$ = 0.001). For subsequent increments the value of strain from the previous increment can be used. From this value, an initial estimate *k*_3_ can be made using Equation (10) and then using Equation (9), an initial value of time, *t*_0_, can be calculated. A Gauss-Newton based iterative method is used to find the value for *ε_i_*_+1_ that minimises the squared error between effective time at the end of the increment, *t*_i+1_, and predicted time, *t_n_*.

(12) $$\varepsilon_{n+1}=\varepsilon_{n}-\frac{f\left(\varepsilon \right)}{f^{\prime }\left(\varepsilon \right)}$$

where

(13) $$f\left(\varepsilon \right)={\left(t_{\text{i+1}}-t_{n}\right)}^{2}$$

Forward difference is used to calculate $f^{\prime }\left(\varepsilon \right)$. A value of $\varepsilon_{i+1}$ is obtained when $\left|t_{i}-t_{n}\right|$ is less than a time error tolerance, TETOL or if the number of iterations, *n*, required to calculate $\varepsilon_{i+1}$ exceeds a set value. Using this value for $\varepsilon_{i+1}$, creep rate, $\dot{\varepsilon }$, can be calculated using Equation (11).

The calculation of creep rate using time hardening is more complex since both $\varepsilon_{i}$ and $\varepsilon_{i+1}$ in Equation (11) cannot be calculated directly. Therefore an iterative method is used to determine $\varepsilon_{i}$ using Equation (12) with

(14) $$f\left(\varepsilon \right)={\left(t_{\text{i}}-t_{n}\right)}^{2}$$

where *t*_i_ is total analysis time at the beginning of the increment. The same iterative method is used to calculate $\varepsilon_{i+1}$ determined using Equations (9) and (10) akin to strain hardening where $t_{\text{i+1}}=t_{\text{i}}+\Delta t$. The formulation for life fraction hardening is similar to that of time fraction hardening however, *t*_i_ is substituted with

(15) $$t_{\text{i,eff}}=w.t_{\text{F}}$$

where *t*_f_ is creep rupture time calculated using Equation (4) and *w* is the life-fraction. *w* is simply $t/t_{\text{f}}$ calculated at the end of each increment based on creep conditions for that increment.

**Figure 21:**
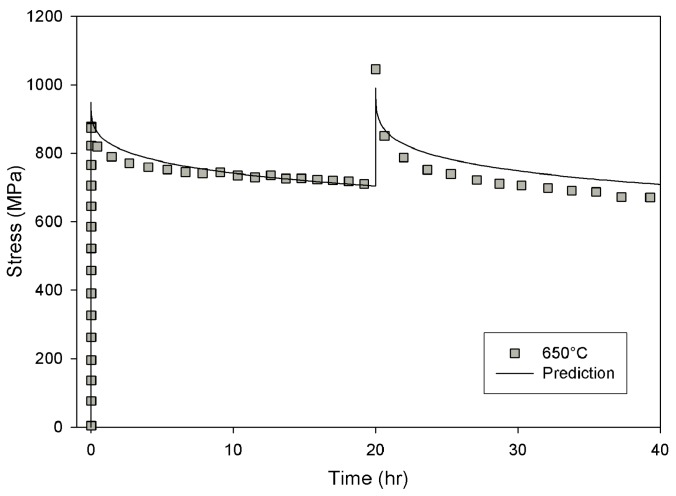
Comparison of predicted and experimentally obtained stress relaxation data.

The method outlined above has been included in user defined material subroutine (*CREEP) for use with Abaqus finite-element analysis software and applied to the prediction of stress relaxation behaviour for the same alloy. An axisymmetric simulation of the full specimen was performed using linear elastic behaviour and isotropic plasticity data obtained from tensile testing at 650 °C. A comparison of predicted and experimentally observed stress relaxation data for Alloy 720Li at 650 °C is shown in Figure 21. It can be seen that the stress relaxation behaviour predicted using the Wilshire based creep model closely resembles test data, providing confidence in this method for describing stress conditions at component features. Clearly, accurate descriptions such as this provide confidence and would impact favourably on the total life prediction of the component. 

## 4. Discussion

The stress and temperature dependence of the creep properties of Alloy 720Li predicted using a traditional power law approach are expressed by the stress exponent *n* and the activation *Q*_c_ in Equation (2). For medium duration tests (250–5000 h) a stress exponent of ~5 was obtained consistent with values obtained for a number of metals and alloys [23]. However, at higher stresses (for shorter duration tests the stress exponent n→14 introducing doubt that Equation (2) can be used to predict creep properties over the full range of test conditions. Furthermore, the activation energy for creep, *Q*_c_, was found to be in excess of 400 kJ mol^−1^ and also varied with test conditions. A lower activation energy for creep, *Q*_c_* = 330 kJ mol^−1^ was found when temperature compensated minimum creep rate, ${\dot{\varepsilon }}_{m}$ was compared to $\sigma /\sigma_{\text{TS}}$ since $\sigma_{\text{TS}}$ varies with test temperature. This value is greater than the activation energy for lattice diffusion in nickel (~280 kJ mol^−1^) and significantly greater than that of grain boundary self diffusion (~115 kJ mol^−1^) [24].

The Wilshire approach extends this concept by relating creep rupture life and minimum creep rate to $-\ln\left(\sigma /\sigma_{\text{TS}}\right)$ using Equations (4) and (5) respectively. This method limits predictions of creep properties over the range $0>\sigma >\sigma_{\text{TS}}$. Using this approach, creep properties for the whole range of test conditions can be predicted over two distinct regimes separated by a break at 0.65*σ*_TS_. At lower stresses values of *u* = 0.184 in Equation (4) and *v* = 0.154 were obtained. Inverting these values gives 1/u≈5.5 and −1/v≈6.5 respectively, closely corresponding to values of *n* for power law creep. At stresses greater than $0.65\sigma_{\text{TS}}$ values of *u* and *v* of 0.0993 and 0.064 respectively were obtained. These values are consistent as σ → σ_TS_, stress levels where power law breakdown is observed.

Previous studies on polycrystalline copper [5], steels [25,26,27] and titanium alloys [11] have shown that a change in slope in Figure 4 and Figure 7 and hence a corresponding changing in the parameters *u*, *v*, *k*1 and *k*2 can be attributed to differences in creep behaviour above and below a level of stress related to yield stress of the alloy. For each creep temperature the 0.2% proof stress $\approx 0.72\sigma_{\text{TS}}$, so it can be assumed that yield occurs at slight lower stresses, consistent with the observed changes in *u* and *v*. Also, analysis of the creep curve shapes at different test conditions gives insight into the mechanisms of creep. The relationship of failure strain, $\varepsilon_{\text{F}}$, on $\sigma /\sigma_{\text{TS}}$ (Figure 12) shows that $\varepsilon_{\text{F}}$ decreases as stress increases, with creep ductilities < 10% at stresses greater than $\approx 0.65\sigma_{\text{TS}}$. Furthermore, Figure 14 shows that $\varepsilon_{\text{p}}$ is negligible below $\approx 0.4\sigma_{\text{TS}}$ but inceases rapidly above $\approx 0.6\sigma_{\text{TS}}$. Since primary creep is characterised by intergranular dislocation work hardening processes it can be assumed that dislocation multiplication occurs within the grains above $\approx 0.6\sigma_{\text{TS}}$. Therefore, subsequent creep must occur in grains with higher dislocation densities resulting in a change in material response consistent with previous studies [5]. At lower stresses, below $\approx 0.6\sigma_{\text{TS}}$, it is assumed that primary creep is limited to slip in suitable orientated grains where local stresses exceed the critical resolved shear stress [28] combined with the movement of pre-existing dislocations within the material under the applied stress.

**Figure 22:**
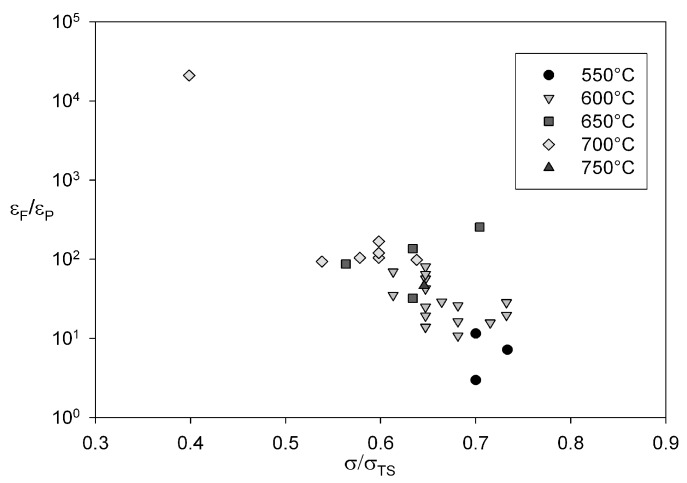
Dependence of $\varepsilon_{\text{F}}/\varepsilon_{\text{P}}$ against normalised stress for Alloy 720Li at 550 °C to 750 °C.

At all applied test conditions Alloy 720Li behaves as a creep-ductile material, since $\varepsilon_{\text{F}}/\varepsilon_{\text{P}}$ ≈1 however this ratio decreases as $\sigma \to \sigma_{\text{TS}}$. Goodall* et al.* [29] proposed that creep-ductile materials are capable of accommodating significant tertiary creep strain prior to failure when $\varepsilon_{\text{F}}/\varepsilon_{\text{P}}\geq 10$. The ratio $\varepsilon_{\text{F}}/\varepsilon_{\text{P}}$ for Alloy 720Li decreases as $\sigma /\sigma_{\text{TS}}$ increases and significantly $\varepsilon_{\text{F}}/\varepsilon_{\text{P}}\approx 10$ when $\sigma /\sigma_{\text{TS}}>0.65$ (Figure 22). Therefore, during creep in the high stress regime, the higher dislocation density results in a higher probability of stress rupture occurring before substantial tertiary creep.

## 5. Conclusions

Minimum creep rate and creep life properties for Alloy 720Li obtained from uniaxial creep testing can be represented using a power law approach (Equation (2)). However, due to the variability of the parameters *A* and *n*, this method is not effective at extrapolating to longer test lives. An Arrhenius term is used to compensate for temperature and if the applied stress is normalised by $\sigma_{\text{TS}}$, the test data can be superimposed onto a single curve.

The Wilshire equations method (Equations (4) and (5)) offers an alternative approach and has been shown to extrapolate to long term data favourably compared to other methods for other alloys. This approach highlights a predictable change in behaviour above and below $0.65\sigma /\sigma_{\text{TS}}$ which is consistent across a range of materials, indicating a change in creep behaviour related to $\sigma_{\text{Y}}$. In applying the equations to the current material it is clear that the creep properties of the material (time to rupture and minimum creep rate behaviour) are well represented by the equations.

Interestingly, values of primary creep strain, ε_p_, were shown to decrease to essentially zero, at $\sigma \approx 0.4\sigma_{\text{TS}}$. Above this stress, but below $\sigma_{\text{Y}}$, it is probable that a limited amount of slip in suitably orientated grains will result in small amounts of primary creep strain. Above $0.65\sigma /\sigma_{\text{TS}}$ it is assumed that dislocation multiplication within the grains during primary creep will cause a change in the bulk creep properties of the material, demonstrated by a change in parameters *u*, *v*, *k*_1_, and *k*_2_ in Equations (4) and (5).

However, the value of the equations in the current application lies not in life extrapolation, but in their ability to represent the holistic creep curve. The Wilshire method has recently been extended to allow predictions of ‘times to strain’ to be made, thus recreating creep curve shapes using Equations (9) and (10). A variation of constants above and below $0.65\sigma /\sigma_{\text{TS}}$ was observed for Equation (9) synonymous with Equations (4) and (5). 

The current method has the advantage of extrapolating favourably with respect to stress and temperature when compared to other reviewed creep methods. A finite element model has been formulated based on this creep curve method, the accuracy of which has been verified against stress relaxation data, the results of which indicate good correlation.
